# Thermophysical analysis of time-dependent magnetized Casson hybrid nanofluid flow (Cu + GO/Kerosene Oil) using Darcy-Forchheimer and thermal radiative models for industrial cooling applications

**DOI:** 10.1038/s41598-025-87743-9

**Published:** 2025-01-27

**Authors:** Amal F. Alharbi, Fida Mohammad, Muhammad Usman, Naseem Khan, Walid Abushiba

**Affiliations:** 1 Department of Mathematics, Faculty of Science, King Abdulaziz University, Jeddah, 21589 Saudi Arabia; 2 Department of Computer Science, Bakhtar University, Kabul, 1001 Afghanistan; 3 Department of Mathematics, City University of Science and Information Technology, Peshawar, 25000 Pakistan; 4 Department of Mathematics, Islamia College University, Peshawar, 25000 Pakistan; 5 College of Engineering, Applied Science University (ASU), Manama, Kingdom of Bahrain

**Keywords:** Casson hybrid nanofluid, Magnetic thermal enhancement, HAM, Stretching sheet, Thermal Radiation, Biomaterials, Nanoscale materials, Materials chemistry

## Abstract

This paper presents an in-depth analytical investigation into the time-dependent flow of a Casson hybrid nanofluid over a radially stretching sheet. The study introduces the effects of magnetic fields and thermal radiation, along with velocity and thermal slip, to model real-world systems for enhancing heat transfer in critical industrial applications. The hybrid nanofluid consists of three nanoparticles—Copper and Graphene Oxide—suspended in Kerosene Oil, selected for their stable and superior thermal properties. The theory of Darcy-Forchheimer, along with the suction and injection effect, is applied to refine the flow behaviour and enhance heat transfer efficiency. The governing nonlinear equations are solved using the Homotopy Analysis Method to provide a robust framework for solution accuracy. The graphical and tabulated results demonstrated that hybrid nanofluid outperforms mono and Casson hybrid nanofluids. The result shows that, at a nanoparticle volume concentration of 0.03, the Casson hybrid nanofluid showed a remarkable 19.99% increase in heat transfer, compared to 14.80% for simple nanofluid. The magnetic parameter and thermal radiation parameter further amplify thermal conductivity. This research provided a critical insight into optimizing thermal management systems for advanced engineering applications, positioning hybrid nanofluid as highly effective solutions for next-generation cooling technologies.

## Introduction

The study of boundary layer flow and heat transfer over stretching sheet has garnered significant interest due to its vital role in industrial, engineering, and medical processes, such as glass fibre production, polymer extrusion, liquid metal handling, heat exchangers, cooling systems, drug delivery systems, and tissue engineering. Efficient heat transfer at the stretching surface is crucial for these applications. Crane^1^ laid the foundation by deriving an exact solution for incompressible viscous flow over a linearly stretching sheet, inspiring extensive research into various geometries and dynamic conditions. Akbar et al.^2^ extended this work to a three-dimensional magnetohydrodynamic non-Newtonian nanofluid, while Ullah et al.^3^ analyzed heat and mass transfer in slip flow over a permeable stretching surface. Siddique et al.^4^ explored the dynamics of a tangent hyperbolic nanofluid with concentration-dependent viscosity and motile microorganisms, and Ali et al.^5^ studied bioconvective slip flow to improve energy systems. Further studies by Nihaal et al.^6^ and Shivaraju et al.^7^ examined MHD Casson hybrid nanofluid flow under thermal radiation and a chemical reaction. Sekhar et al.^8^ analyzed viscoelastic nanofluid over a stretching sheet with slip condition, while Prakash et al.^9^ investigated hybrid nanofluid on an exponentially stretching surface under heat source effect. Axisymmetric flow over radially stretching a sheet has also been extensively studied, with Shah et al.^10^ focusing on two immiscible fluids under a magnetic field, Bhatti et al.^11^ investigating convection flow in a porous microchannel, and Umavathi et al.^12^ exploring radiative heat transfer in squeezing flow. Recent work has addressed time-dependent behaviour, including Azar et al.^13^ on porous wedge, Ali et al.^14^ on cross Casson hybrid nanofluid with entropy minimization, Abbas et al.^15^ on radiative Sutterby nanofluid, and Ullah et al.^16^ on MHD Casson hybrid nanofluid with nanoparticle shape. While mono and hybrid nanofluid have been extensively analyzed, the behaviour of Casson hybrid nanofluid in these geometries remains underexplored, motivating the present study.

Casson hybrid nanofluid (HNF), formed by dispersing two distinct nanoparticles in a base fluid, offered superior thermal and rheological properties compared to conventional nanofluid or base fluid alone. These properties make HNF highly suitable for applications in automotive radiators, microelectronics cooling, solar energy systems, and nuclear reactors. For instance, Madhukesh et al.^17^examined the thermal and flow behaviour of HNF over an extending surface, reporting enhanced heat transfer due to improved nanoparticle synergy. Similarly, Nihaal and Mahabaleshwar^18^ demonstrated significant thermal efficiency improvement with optimized volume fraction in HNF, highlighting their effectiveness in an advanced heat transfer system. Karthik et al.^19^ further investigated HNF embedded in porous media and observed enhanced thermal conductivity and efficient energy transport, making them ideal for a high-performance cooling system. In contrast, Casson HNF, composed of two types of nanoparticles, present even greater enhancements in thermal and rheological properties, making them ideal for advanced heat transfer applications like cooling automotive engines, solar collectors, and microelectronics. Studies on HNF, such as Cu + Al2O3 in water, have demonstrated superior thermal efficiency and momentum flow compared to both mono and Casson HNF. Additionally, Nasir et al.^20^ confirmed that HNF composition significantly enhanced thermal conductivity and energy transport, making HNFs an emerging focus for advanced cooling systems and high-efficiency thermal management applications.

This study analytically investigated the flow of Casson hybrid nanofluid over a radially stretching sheet, focusing on the effect of magnetic field, thermal radiation, and slip condition. Adnan et al.^21^ highlighted the role of magnetic field and radiation in enhancing heat transfer in hybrid nanofluid, while Khan et al.^22^ demonstrated that multiple slip conditions and thermal radiation significantly improve heat and mass transfer. The impact of suction and injection on flow characteristics is examined using the theory of Darcy-Forchheimer, building on the finding of Panda et al.^23^ and Kumari et al.^24^. Their results showed the optimization potential of a suction parameter. Additionally, Khan et al.^25^ and Sekhar et al.^8^ explored the effect of thermal radiation and a heat source on nanofluid flow, emphasizing their importance in thermal management. The homotopy analysis method (HAM) is employed to solve the governing equations, following the approach of Sohail et al.^26^, who validated its effectiveness in analyzing unsteady dynamics over a stretching sheet. This study underscores the potential of hybrid nanofluids in enhancing thermal efficiency for industrial applications.

This study advances the understanding of Casson hybrid nanofluid behaviour by exploring its time-dependent flow dynamics over a radially stretching sheet, particularly under the influence of magnetic fields, thermal radiation, and slip conditions. While related work exists in the literature, this research fills a critical gap by extending the Darcy-Forchheimer theory to include suction and injection effects, thereby providing a more comprehensive analysis of flow behaviour and heat transfer efficiency. The novelty lies in demonstrating the superior thermal performance of Casson hybrid nanofluids with copper and graphene oxide nanoparticles in kerosene oil, achieving a remarkable 19.99% increase in heat transfer at a nanoparticle concentration of 0.03. This work raises key questions, such as: How do magnetic and thermal radiation parameters amplify thermal conductivity? What specific roles do suction and injection play in optimizing the thermal management of advanced cooling technologies? These insights aim to guide future research and industrial applications in next-generation heat transfer systems.

**Fig. 1:**
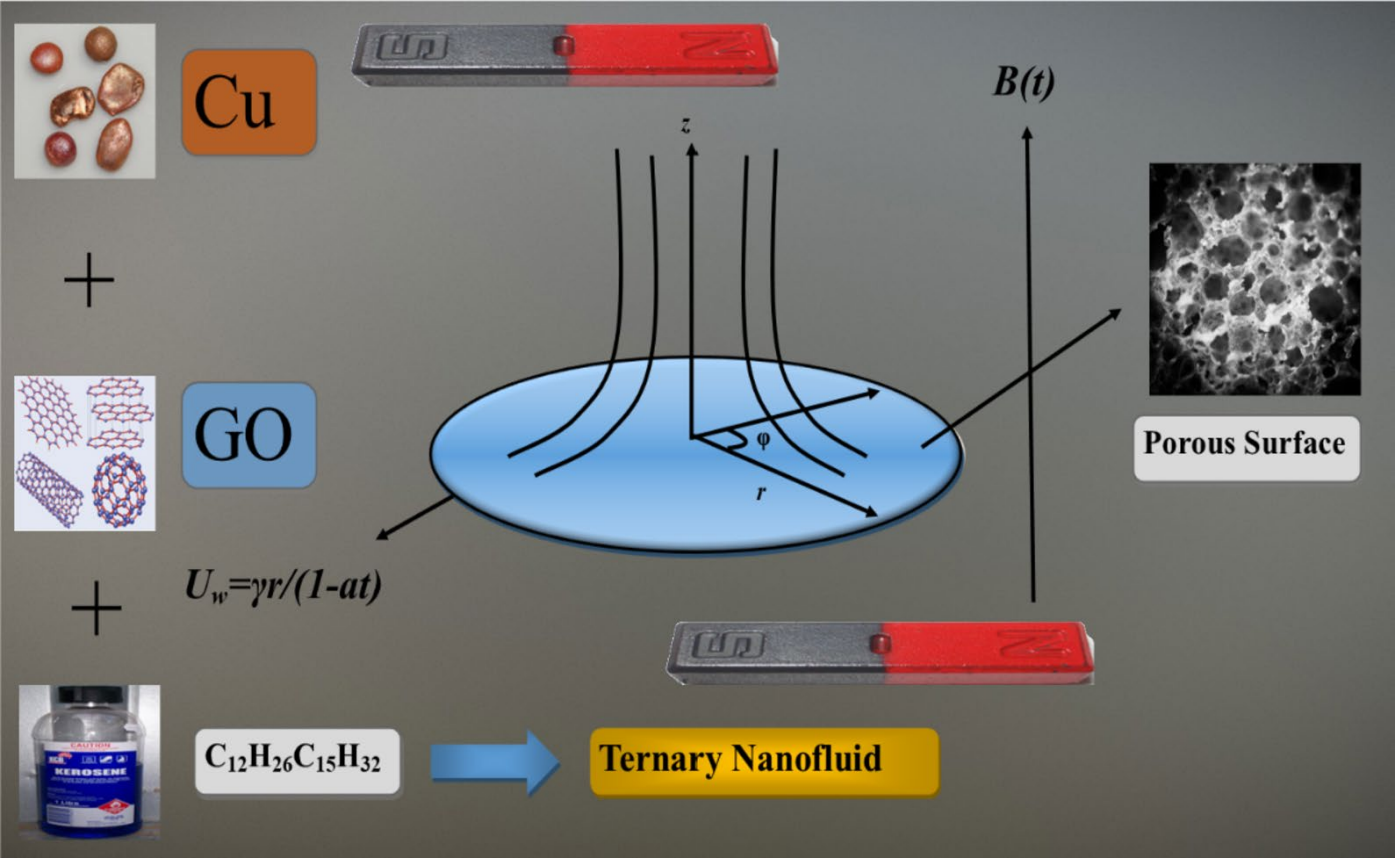
Geometry of the problem.

## Mathematical formulation

Consider an unsteady two-dimensional and laminar flow of Casson hybrid nanofluid over a stretching surface as shown in Fig. 1. The configuration consists of a sheet positioned at *z = 0*, which occupies the space *z > 0*. A cylindrical polar coordinate system *(r*,* θ*,* z)* was utilized for computational analysis. *T (r*,* z*,* t)* represents the temperature, indicating its dependence on radial position (*r*), axial position (*z*), and time (*t*). The velocity field [v (*r*,* z*,* t*)] is a vector containing radial (*u*) and axial (*w*) components. The fluid’s temperature takes the form $${T_w}={T_\infty }+{\raise0.7ex\hbox{${br}$} \!\mathord{\left/ {\vphantom {{br} {1 - at}}}\right.\kern-0pt}\!\lower0.7ex\hbox{${1 - at}$}}$$. Here T_*w*_ denotes the wall temperature, while _*∞*_denotes the far-field temperature^3,27–29^.

$$\frac{{\partial u}}{{\partial r}}+\frac{u}{r}+\frac{{\partial w}}{{\partial z}}=0$$

$$\frac{{\partial u}}{{\partial t}}+u\frac{{\partial u}}{{\partial r}}+w\frac{{\partial u}}{{\partial z}}=\frac{{{\mu _{hnf}}}}{{{\rho _{hnf}}}}\left( {1+\frac{1}{\beta }} \right)\frac{{{\partial ^2}u}}{{\partial {z^2}}} - \frac{{{\sigma _{hnf}}{B_0}{{\left( t \right)}^2}u}}{{{\rho _{hnf}}}} - {F_o}{u^2} - \frac{{{\upsilon _{hnf}}}}{{{k_o}}}u$$

$$\frac{{\partial T}}{{\partial t}}+u\frac{{\partial T}}{{\partial r}}+w\frac{{\partial T}}{{\partial z}}=\frac{{{k_{hnf}}}}{{{{\left( {\rho {c_p}} \right)}_{hnf}}}}\frac{{{\partial ^2}T}}{{\partial {z^2}}} - \frac{1}{{{{(\rho {c_p})}_{hnf}}}}\frac{{\partial {q_r}}}{{\partial z}}+\frac{{{\mu _{hnf}}}}{{{{\left( {\rho {c_p}} \right)}_{hnf}}}}{\left( {\frac{{\partial u}}{{\partial z}}} \right)^2}+\frac{{{\sigma _{hnf}}{B_0}{{\left( t \right)}^2}{u^2}}}{{{{\left( {\rho {c_p}} \right)}_{hnf}}}}$$

As we expand ${q_r}$ using the Taylor series and disregarding higher-order terms, it can be expressed in the following manner.

$${q_r}= - \frac{{4{\sigma ^*}}}{{3{k^*}}}\frac{{{\partial ^4}T}}{{\partial {z^4}}},{\text{ }}{\tilde {T}^4} \approx 4T_{\infty }^{3} - 3T_{\infty }^{4},$$

By incorporating this term into the energy equation, we derive the following outcome.

$$\frac{{\partial T}}{{\partial t}}+u\frac{{\partial T}}{{\partial r}}+w\frac{{\partial T}}{{\partial z}}=\left( {\frac{{{k_{hnf}}}}{{{{\left( {\rho {c_p}} \right)}_{hnf}}}}+\frac{{16{\sigma ^*}T_{\infty }^{3}}}{{3{k^*}{{(\rho {c_p})}_{hnf}}}}} \right)\frac{{{\partial ^2}T}}{{\partial {z^2}}}+\frac{{{\mu _{hnf}}}}{{{{\left( {\rho {c_p}} \right)}_{hnf}}}}{\left( {\frac{{\partial u}}{{\partial z}}} \right)^2}+\frac{{{\sigma _{hnf}}{B_0}{{\left( t \right)}^2}{u^2}}}{{{{\left( {\rho {c_p}} \right)}_{hnf}}}}$$

The boundary conditions near and distant from the sheet are:

$$u={U_w}+{U_{slip}},w={W_0}= - 2\Lambda \sqrt {\frac{{{U_w}{\upsilon _f}}}{r}} ,T={T_w}+{T_{slip}}$$ at $$z=0$$

$$u \to {u_\infty },T \to {T_\infty }$$ as $$z \to \infty$$

The sheet undergoes radial expansion with a surface velocity ${U_w}={{\gamma r} \mathord{\left/ {\vphantom {{\gamma r} {1 - at}}} \right. \kern-0pt} {1 - at}}$, and stream velocity ${u_\infty }$, the variables *γ* and *a* indicate parameters that describe the physical properties of the stretching surface. The term ${U_{slip}}={N_1}\frac{{\partial u}}{{\partial z}}$ and ${T_{slip}}={N_2}\frac{{\partial T}}{{\partial z}}$ is represented the slip velocity and slip temperature respectively with the hydraulic slip ${N_1}$ and thermal slip ${N_2}$ factors. Here the surface ${W_0}>0\left( {\Lambda <0} \right)$ represents injection and ${W_0}<0\left( {\Lambda>0} \right)$ represents suction. A magnetic field with a strength of ${B_0}\left( t \right)={{{B_0}} \mathord{\left/ {\vphantom {{{B_0}} {\sqrt {1 - at} }}} \right. \kern-0pt} {\sqrt {1 - at} }}$ acts perpendicular to the stretching sheet. This model considers the impacts of suction, joule heating, and viscous dissipation. Also adopting the Darcy–Forchheimer concept to the flow model, the permeability of the porous medium is represented by *k*_*0*_, and ${{{F_0}={C_b}} \mathord{\left/ {\vphantom {{{F_0}={C_b}} {r\sqrt {{k_o}} }}} \right. \kern-0pt} {r\sqrt {{k_o}} }}$ is the Forchheimer number, where *C*_*b*_ is the drag coefficient. The experimental values of the nanoparticles and base fluid are presented in Table 1. The thermophysical properties of the ternary nanofluid model are described in Table 2 as can be seen.

**Table 1:** Organizes the Thermophysical properties of copper, graphene oxide, and kerosene oil Agarwal et al.^30^.

Physical characters	Copper Cu	Graphene oxide GO	Kerosine Oil C_12_H_26_C_15_H_32_
Thermal conductivity $k\left( {W/mK} \right)$	765	5000	0.145
Electrical conductivity $\sigma \left( {S/m} \right)$	5.96*10^7^	6.30*10^7^	21*10^−6^
Specific heat capacity ${C_p}\left( {J/kgK} \right)$	531.8	717	2090
Density $\rho \left( {kg/{m^3}} \right)$	632	1800	783
Prandtl number $\left( {\Pr } \right)$	--	--	6.135

The method of similarity transformation applied in this study is demonstrated as follows

$$\psi =\frac{{ - {r^2}{U_w}}}{{\sqrt {\operatorname{Re} } }}f\left( \eta \right)$$, $$\eta =\frac{z}{r}\sqrt {\operatorname{Re} }$$ and $$T=\Theta \left( \eta \right)\left( {{T_w} - {T_\infty }} \right)+{T_\infty }$$

The stream function () now represents the velocity components in both the longitudinal and transverse directions.

$$u=\frac{1}{r}\frac{{\partial \psi }}{{\partial z}}{\text{ }},w=\frac{1}{r}\frac{{\partial \psi }}{{\partial r}}.$$

This satisfies the continuity equation.

Similarity transformations are applied to convert the momentum and energy equations into a nondimensional form.

$$\frac{{{\upsilon _{hnf}}}}{{{\upsilon _f}}}\left( {\left( {1+\frac{1}{\beta }} \right)f^{\prime\prime\prime} - \frac{1}{{\lambda \cdot \operatorname{Re} }}f^{\prime}} \right)+2f\cdot f^{\prime\prime} - \left( {1+Fr} \right){f^{\prime {2}}} - S\left( {f^{\prime}+\frac{\eta }{2}f^{\prime\prime}} \right) - \frac{{{{{\sigma _{hnf}}} \mathord{\left/ {\vphantom {{{\sigma _{hnf}}} {{\sigma _f}}}} \right. \kern-0pt} {{\sigma _f}}}}}{{{{{\rho _{hnf}}} \mathord{\left/ {\vphantom {{{\rho _{hnf}}} {{\rho _f}}}} \right. \kern-0pt} {{\rho _f}}}}}{M_g}f^{\prime}=0$$

$$\frac{1}{{{\operatorname{P} _r}}}\left( {\frac{{{k_{hnf}}}}{{{k_f}}}+\frac{4}{3}Rd} \right)\Theta ^{\prime\prime}+\frac{{{{\left( {\rho {c_p}} \right)}_{hnf}}}}{{{{\left( {\rho {c_p}} \right)}_f}}}\left( \begin{gathered} {\text{ }}2f\Theta ^{\prime} \hfill \\ - \frac{\eta }{2}S\Theta ^{\prime} \hfill \\ \end{gathered} \right)+\frac{{{\mu _{hnf}}}}{{{\mu _f}}}{E_c}{f^{\prime\prime{2}}}+\frac{{{{{\sigma _{hnf}}} \mathord{\left/ {\vphantom {{{\sigma _{hnf}}} {{\sigma _f}}}} \right. \kern-0pt} {{\sigma _f}}}}}{{{{{{\left( {\rho {c_p}} \right)}_{hnf}}} \mathord{\left/ {\vphantom {{{{\left( {\rho {c_p}} \right)}_{hnf}}} {{{\left( {\rho {c_p}} \right)}_f}}}} \right. \kern-0pt} {{{\left( {\rho {c_p}} \right)}_f}}}}}{M_g}{E_c}{f^{\prime\prime{2}}}=0$$

The boundary condition (4) now transforms into.

$f^{\prime}=1+{l_1}f^{\prime\prime},{\text{ }}f={S_p},{\text{ }}\Theta =1+{l_2}\Theta ^{\prime}$ at $\eta =0$ and $f^{\prime} \to 0,{\text{ }}\Theta \to 0$ as $\eta \to \infty$

## Dimensionless parameters

$$\begin{gathered} S=\frac{a}{\gamma },{\text{ }}M=\frac{{{\sigma _f}B_{0}^{2}}}{{\gamma {\rho _f}}},{\text{ }}Fr=\frac{{{c_b}}}{{\sqrt {{k_0}} }},{\text{ }}\lambda =\frac{{{k_0}}}{{{r^2}}},{\text{ }}\operatorname{Re} =\frac{{r{U_w}}}{{{\upsilon _f}}},{\text{ }}Ec=\frac{{U_{w}^{2}}}{{\left( {{T_w} - {T_\infty }} \right){c_p}}},{\text{ }}Rd=\frac{{4{\delta ^*}{T^3}}}{{3{k_f}{k^*}}}, \hfill \\ {\text{ }}\Pr =\frac{{{\alpha _f}}}{{{\upsilon _f}}},{l_1}={N_1}\sqrt {\frac{{{U_w}}}{{r{\upsilon _f}}}} ,{\text{ }}{l_2}={N_2}\sqrt {\frac{{{U_w}}}{{r{\upsilon _f}}}} . \hfill \\ \end{gathered}$$

**Table 2:** The thermophysical properties of Casson hybrid Nanofluid Bilal et al.^31^ and Kumar et al.^32^.

Property	Model
Dynamic Viscosity	$\frac{{{\mu _{hnf}}}}{{{\mu _f}}}={\left( {1 - {\phi _1}} \right)^{ - 2.5}}{\left( {1 - {\phi _2}} \right)^{ - 2.5}}$
Density	$\frac{{{\rho _{hnf}}}}{{{\rho _f}}}=\left( {1 - {\phi _1}} \right)\left[ {\left( {1 - {\phi _2}} \right)+\frac{{{\rho _2}}}{{{\rho _f}}}{\phi _2}} \right]+\frac{{{\rho _1}}}{{{\rho _f}}}{\phi _1}$
Heat Capacity	$\frac{{\rho {c_{hnf}}}}{{\rho {c_f}}}=\left( {1 - {\phi _1}} \right)\left[ {\left( {1 - {\phi _2}} \right)+\frac{{\rho {c_2}}}{{\rho {c_f}}}{\phi _2}} \right]+\frac{{\rho {c_1}}}{{\rho {c_f}}}{\phi _1}$
Thermal Conductivity	${k_{hnf}}={k_{nf}}\frac{{{k_1}+2{k_{nf}} - 2{\phi _1}\left( {{k_{nf}} - {k_1}} \right)}}{{{k_1}+2{k_{nf}}+{\phi _1}\left( {{k_{nf}} - {k_1}} \right)}}$
Electrical Conductivity	${\sigma _{hnf}}={\sigma _{nf}}\left[ {1+\frac{{3\left( {{{{\sigma _1}} \mathord{\left/ {\vphantom {{{\sigma _1}} {{\sigma _{nf}}}}} \right. \kern-0pt} {{\sigma _{nf}}}} - 1} \right){\phi _1}}}{{\left( {{{{\sigma _1}} \mathord{\left/ {\vphantom {{{\sigma _1}} {{\sigma _{nf}}}}} \right. \kern-0pt} {{\sigma _{nf}}}}+2} \right) - \left( {{{{\sigma _1}} \mathord{\left/ {\vphantom {{{\sigma _1}} {{\sigma _{nf}}}}} \right. \kern-0pt} {{\sigma _{nf}}}} - 1} \right){\phi _1}}}} \right]$
Kinematic Viscosity	${\upsilon _{hnf}}=\frac{{{{{\mu _{hnf}}} \mathord{\left/ {\vphantom {{{\mu _{hnf}}} {{\mu _f}}}} \right. \kern-0pt} {{\mu _f}}}}}{{{{{\rho _{hnf}}} \mathord{\left/ {\vphantom {{{\rho _{hnf}}} {{\rho _f}}}} \right. \kern-0pt} {{\rho _f}}}}}$
Thermal Diffusivity	${\alpha _{hnf}}=\frac{{{{{k_{hnf}}} \mathord{\left/ {\vphantom {{{k_{hnf}}} {{k_f}}}} \right. \kern-0pt} {{k_f}}}}}{{{{{{\left( {\rho {c_p}} \right)}_{hnf}}} \mathord{\left/ {\vphantom {{{{\left( {\rho {c_p}} \right)}_{hnf}}} {{{\left( {\rho {c_p}} \right)}_f}}}} \right. \kern-0pt} {{{\left( {\rho {c_p}} \right)}_f}}}}}$

## Physical quantities

Local shear rate (*C*_*f*_) and Nusselt number (*Nu*) are the key physical factors that determine the flow challenge, denoted as $${C_f}={\raise0.7ex\hbox{${{\tau _w}}$} \!\mathord{\left/ {\vphantom {{{\tau _w}} {{\rho _f}U_{w}^{2}}}}\right.\kern-0pt}\!\lower0.7ex\hbox{${{\rho _f}U_{w}^{2}}$}}\;$$ & $$Nu={\raise0.7ex\hbox{${r{q_w}}$} \!\mathord{\left/ {\vphantom {{r{q_w}} {{k_f}\left( {{T_w} - {T_\infty }} \right)}}}\right.\kern-0pt}\!\lower0.7ex\hbox{${{k_f}\left( {{T_w} - {T_\infty }} \right)}$}}$$, respectively, where $${\tau _w}={\mu _{hnf}}\frac{{\partial u}}{{\partial z}}$$ & $${q_w}= - \left( {{k_{hnh}}+\frac{3}{4}Rd} \right)\frac{{\partial T}}{{\partial z}}$$ at $$z=0$$. The values of the shear rate coefficient and heat flow at $$z=\infty$$ can be simplified in non-dimensional form as $${C_f}\sqrt {\operatorname{Re} } ={\raise0.7ex\hbox{${{\mu _{hnf}}}$} \!\mathord{\left/ {\vphantom {{{\mu _{hnf}}} {{\mu _f}}}}\right.\kern-0pt}\!\lower0.7ex\hbox{${{\mu _f}}$}}f^{\prime\prime}\left( 0 \right)$$ & $$Nu{\operatorname{Re} ^{ - {1 \mathord{\left/ {\vphantom {1 2}} \right. \kern-0pt} 2}}}=\left( {\frac{{{k_{hnf}}}}{{{k_f}}}+\frac{3}{4}Rd} \right)\Theta ^{\prime}\left( 0 \right)$$ .

## Solution methodology

Introducing the method for solving considered nonlinear problems called the homotopy analysis method (HAM), created by Professor Shijun Liao^33^. This technique uses the topological idea of homotopy to produce a convergent series solution for nonlinear systems. The system of ordinary differential equations that the series solutions come from was created using the HAM approach. Linear operators ${L_1}\& {L_2}$ and initial guesses ${f_0}\left( \eta \right)\& {\Theta _0}\left( \eta \right)$ are taken in the form given following:

$${L_1}=f^{\prime\prime\prime}+f^{\prime\prime},{\text{ }}{L_2}=\Theta ^{\prime\prime}+\Theta ^{\prime}$$

$${f_0}\left( \eta \right)=\frac{{1 - {{\text{e}}^{ - x}}+{S_p}+{l_1}{S_p}}}{{1+{l_1}}},{\text{ }}\& {\text{ }}{\Theta _0}\left( 0 \right)=\frac{{{{\text{e}}^{ - x}}}}{{1+{l_2}}}.$$

Subject to the following properties

26 $${L_1}\left[ {{D_1}} \right]=0,{\text{ }}{L_2}\left[ {{D_2}{e^\eta }+{D_3}{e^{ - \eta }}} \right]=0{\text{ }}\& {L_3}\left[ {{D_4}{e^\eta }+{D_5}{e^{ - \eta }}} \right]=0$$

In which ${D_i}\left( {i=1 - 5} \right)$ is the constant. The problems have been constructed for zeroth and m^th^ order distortion as given the above linear operators. The problems have been resolved with the help of Mathematica software.

## Results validation/ HAM convergence

To verify our results, the convergence domain and the pace of homotopic solutions of ternary hybrid nanofluid flow are regulated by the non-zero convergence control parameters (CCPs) ${h_f}$and ${h_\Theta }$. We have used Liao’s definition of depreciation by average mean square errors^31^ to get the ideal value of CCPs.

31 $$\varepsilon _{m}^{f}=\frac{1}{{k+1}}{\sum\limits_{{f=0}}^{k} {\left[ {{N_f}{{\left( {\sum\limits_{{i=0}}^{m} {f\left( \zeta \right)} } \right)}_{\zeta =j\delta \zeta }}} \right]} ^2},$$

32 $$\varepsilon _{m}^{\Theta }=\frac{1}{{k+1}}{\sum\limits_{{f=0}}^{k} {\left[ {{N_\Theta }{{\left( {\sum\limits_{{i=0}}^{m} {f\left( \zeta \right)} ,\,\,\,\sum\limits_{{i=0}}^{m} {\Theta \left( \zeta \right)} } \right)}_{\zeta =j\delta \zeta }}} \right]} ^2}.$$

Following Liao:

33 $$\varepsilon _{m}^{t}=\,\,\varepsilon _{m}^{f}+\,\varepsilon _{m}^{\Theta }.$$

**Fig. 2:**
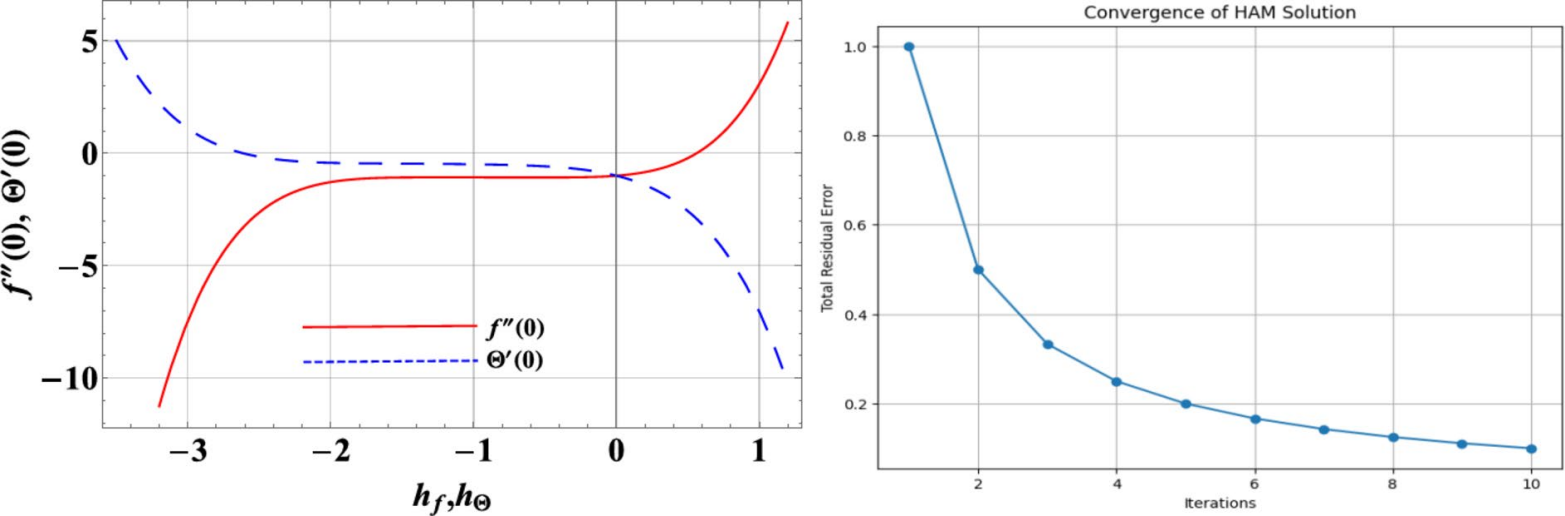
(**a**-**b**) Optimal control convergence parameter curves for ${h_f}\& {h_\Theta }$ and total residual error.

Where $\varepsilon _{m}^{t}$stands for total squared residual error, $\delta \zeta =0.5$ and $m=10$the optimal values of CCPs at 2nd order of approximations for the hybrid nanofluid case are ${h_f}\varepsilon \left[ { - 2,0.5} \right]$ and ${h_\Theta }\varepsilon \left[ { - 1.7,0.4} \right]$ as shown in Fig. 2(a). The total squared residual error for the HAM solution is shown in Fig. 2(b), while Individual mean squared residual errors. Also, for better accuracy, Table 3 represents the comparative analysis of $f^{\prime\prime}\left( 0 \right)$ against M with the existence literature [34, 35, 36].

**Table 3:** Comparison values of $f^{\prime\prime}\left( 0 \right)$ for different values of M when ${\phi _1},{\phi _2},{\phi _3}=0$.

M	$f^{\prime\prime}\left( 0 \right)$			
	Turkyilmazoglu^34^	Kumar et al^35^.	Hayat et al. ^36^	Present
0.0	1	1	1	1
0.2	0.91287	0.9131	0.912871	0.912905
0.4	0.84516	0.8452	0.845154	0.845083
0.6	0.70632	0.7047	0.70638	0.705843

## Graphical and tabulated results and discussions

In this section, we presented and discussed the graphical results derived from the power series solution of the governing ordinary differential equations (ODEs) subject to the prescribed boundary conditions, using the Homotopy Analysis Method (HAM). The iterative power series solutions obtained through HAM are utilized to explore the effects of various dimensionless parameters on the fluid flow and heat transfer profiles.

**Fig. 3:**
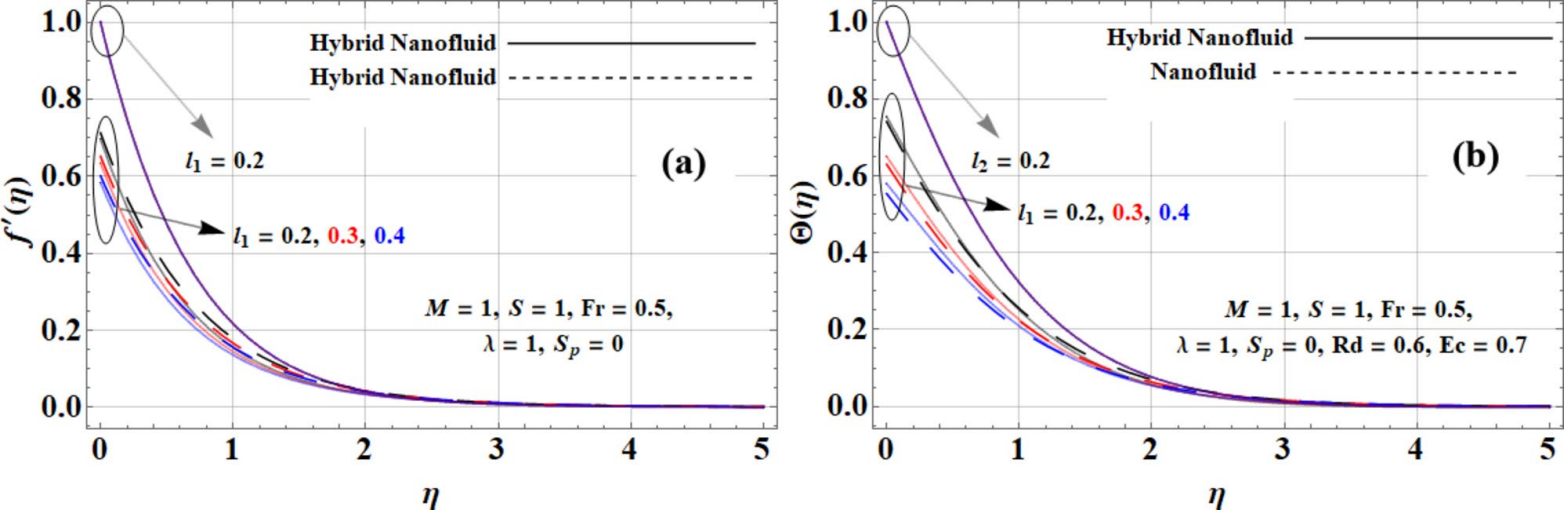
Slip impact on **(a)** velocity and **(b)** temperature profiles.

The effect of velocity and thermal slip can be seen in Fig. 3(a-b) against velocity and temperature profiles. The velocity slip parameter (*l*_*1*_ *= 0.2*,* 0.3*,* 0.4*) decreases the velocity profile as the slip increases, reducing the interaction between the fluid and the surface. No further effect is observed when *l*_*1*_ *= 0*. The nanofluid (*Cu + kerosene oil*) has the highest velocity due to copper’s high thermal conductivity (*765 W/m·K*). The Casson hybrid nanofluid (*Cu + GO + kerosene oil*) shows the lowest velocity due to the higher density and specific heat capacity of graphene oxide (*GO: 5000 W/m·K*,* 1800 kg/m³*,* 717 J/kg·K*), causing greater inertia. Conversely, for temperature profiles, the thermal slip parameter decreases the temperature as it limits heat transfer, with the Casson hybrid nanofluid showing the highest temperature due to *GO*’s superior heat retention, followed by the Casson hybrid nanofluid and the nanofluid, which has the lowest temperature owing to copper’s lower specific heat capacity (*531.8 J/kg·K*).

**Fig. 4:**
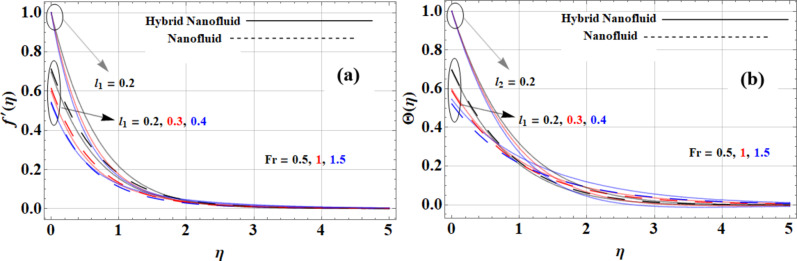
Forchheimer parameter impact on **(a)** velocity and **(b)** temperature profiles.

Figure 4(a-b) shows the Forchheimer parameter influences the behaviour of nanofluids, Casson hybrid nanofluids, and Casson hybrid nanofluids, particularly regarding velocity and temperature profiles under both slip and no-slip conditions. For the *Cu + Kerosene Oil* nanofluid, the high thermal conductivity of copper (*765 W/m·K*) facilitates effective heat transfer, yet the low thermal conductivity of kerosene oil (*0.145 W/m·K*) may lead to substantial temperature gradients, especially under no-slip conditions. As the Forchheimer parameter (*Fr*) increases, inertial effects become more pronounced, resulting in flatter velocity profiles and reduced velocity gradients. The *Cu + GO + Kerosene Oil* Casson hybrid nanofluid, leveraging the exceptionally high thermal conductivity of graphene oxide (*5000 W/m·K*), exhibits improved heat distribution and reduced temperature gradients, particularly under slip conditions, where the fluid can move more freely along the boundaries. Overall, as the Forchheimer parameter increases, both slip and no-slip conditions enhance the fluid’s thermal efficiency, with higher thermal conductivity materials leading to better heat transfer and smoother velocity profiles in applications involving fluid flow in porous media.

**Fig. 5:**
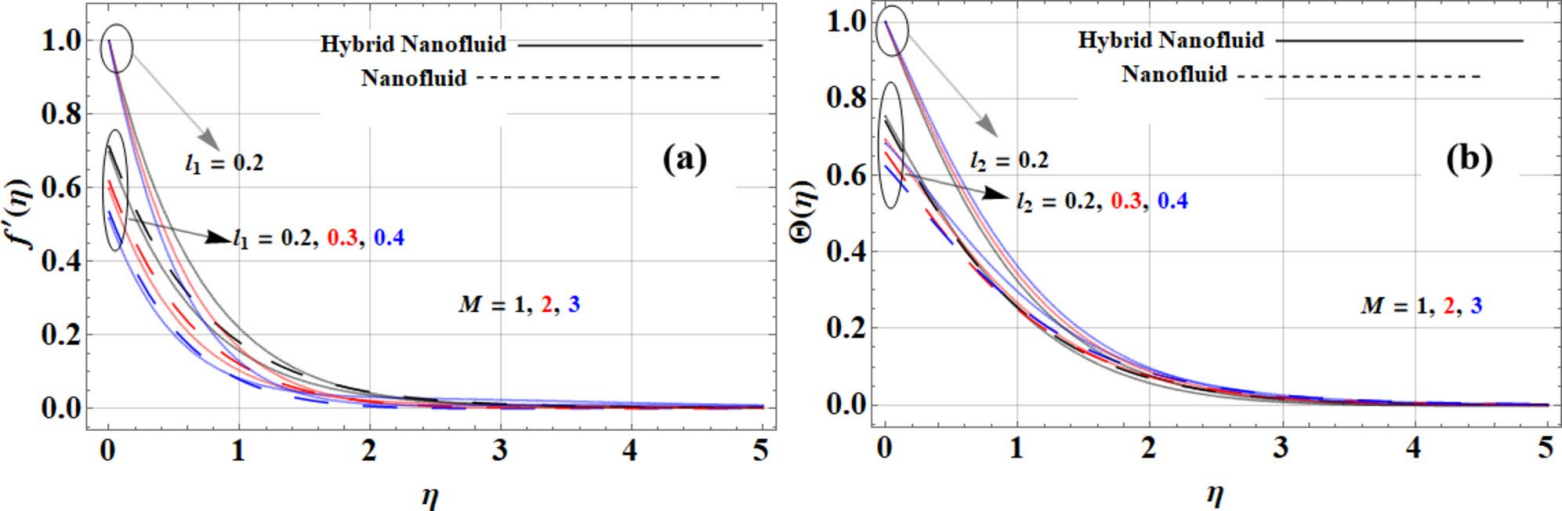
Magnetic parameter impact on **(a)** velocity and **(b)** temperature profiles.

The presence of a magnetic field influences the velocity and temperature profiles of nanofluids, with notable differences across various compositions as shown in Fig. 5(a-b). As the magnetic parameter increases, the velocity profile generally decreases due to the resistance posed by Lorentz forces, leading to flatter velocity distributions, especially under no-slip conditions. Conversely, this magnetic field enhances thermal mixing, resulting in increased temperature profiles across the fluid. The Casson hybrid nanofluid (*Cu + GO + Kerosene Oil*) demonstrates the best temperature performance due to the high thermal conductivity of graphene oxide (*5000 W/m·K*), which facilitates effective heat transfer and uniform temperature distributions. In contrast, the *Cu + Kerosene Oil* nanofluid exhibits superior velocity performance, as the high conductivity of copper (*765 W/m·K*) allows it to maintain better flow characteristics despite the magnetic influence. Thus, while the magnetic field dampens the velocity profiles across all nanofluids, it paradoxically enhances the temperature profiles, showcasing the Casson hybrid nanofluid’s effectiveness in temperature uniformity and the copper-based nanofluid’s advantage in velocity performance.

**Fig. 6:**
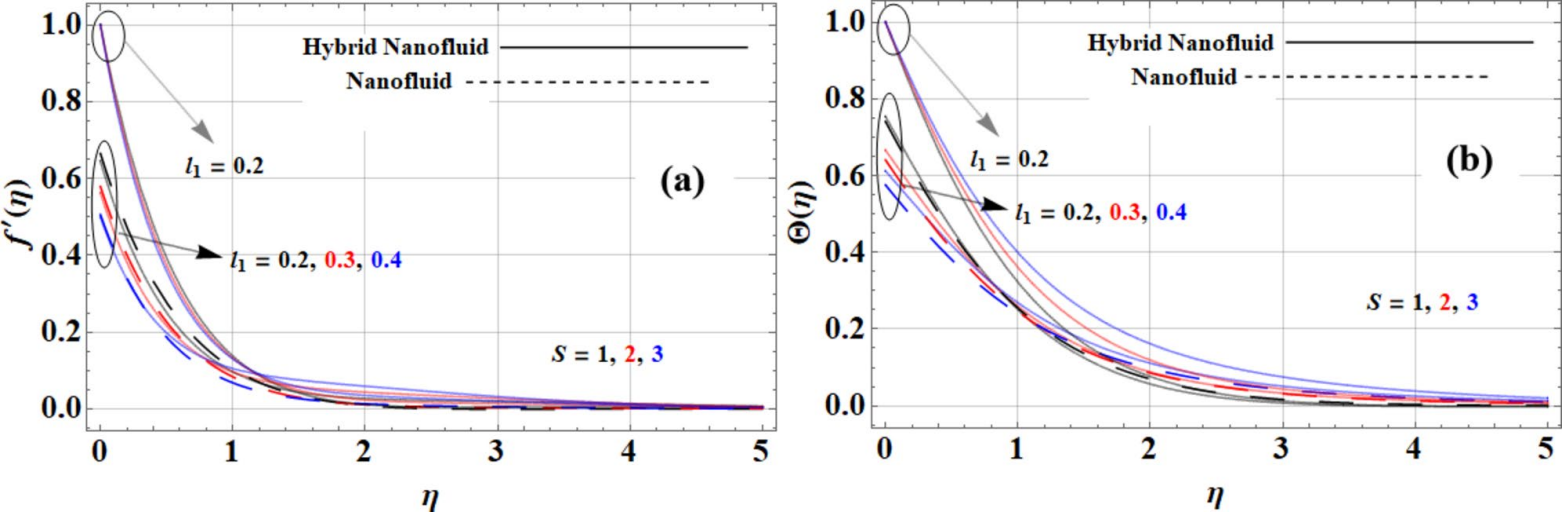
Unsteady parameter impact on **(a)** velocity and **(b)** temperature profiles.

The unsteady parameter negatively impacts both the velocity and temperature profiles of nanofluids under slip and no-slip conditions. This scenario is captured in Fig. 6(a-b) as can be seen. The time-dependent effects the evident as the unsteady parameter increases, which brings a decrease in the velocity profile due to increased inertia and viscous damping forces. This force hinders fluid motion This reduction is evident in both slip and no-slip conditions. The temperature profile also decreases, as the unsteady effects disrupt the thermal equilibrium and mixing within the fluid. While the unsteady parameter decreases both velocity and temperature profiles, the Casson hybrid nanofluid excels in maintaining temperature uniformity, whereas the copper-based nanofluid demonstrates superior velocity performance under these dynamic conditions.

**Fig. 7:**
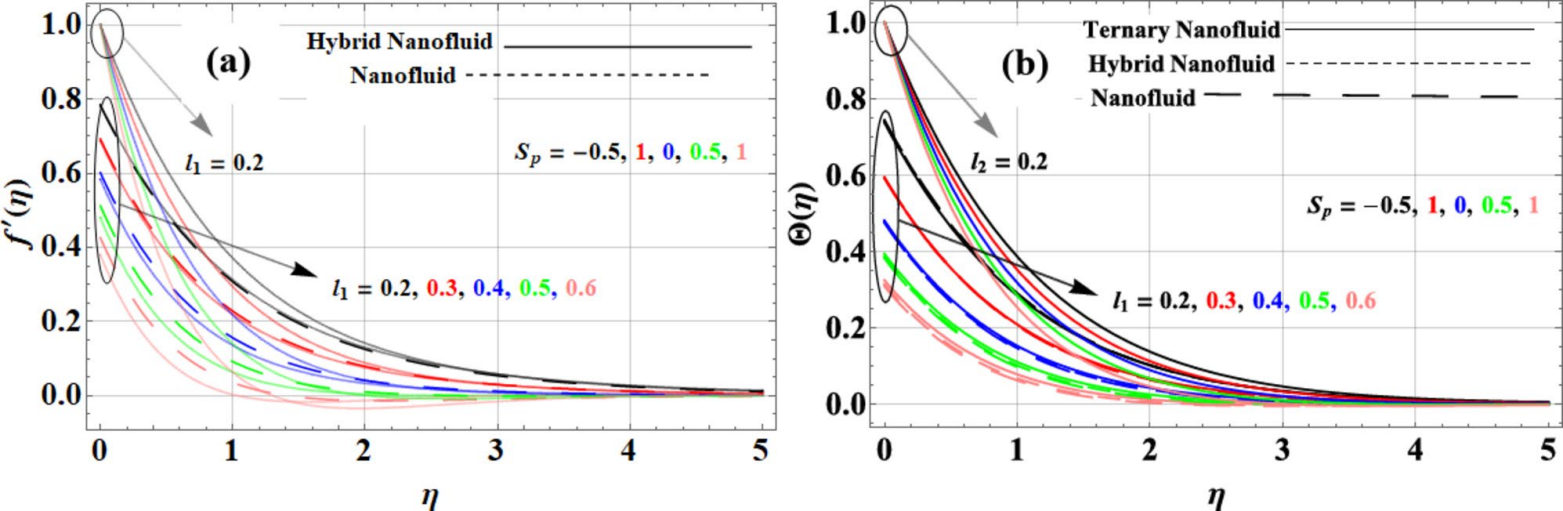
Suction/injection parameter impact on **(a)** velocity and **(b)** temperature profiles.

As shown in Fig. 7(a-b), the suction/injection parameter affects the velocity and temperature profiles in both slip and no-slip scenarios. Applying suction improves the velocity profile by pushing the fluid in the direction of the boundary. With a density of 632 kg/m³ for copper and 783 kg/m³ for kerosene oil, the Cu + Kerosene Oil nanofluid benefits most from this effect, allowing the fluid to sustain a greater velocity. A more uniform but lower velocity profile can result from the injection’s tendency to lower the velocity profile, which can also damper the current flow. Suction and injection have an important influence on temperature profiles as well. Suction enhances the temperature profile by promoting better mixing and reducing thermal gradients, particularly in the Casson hybrid nanofluid (Cu + GO + Kerosene Oil), due to the superior thermal conductivity of graphene oxide (5000 W/m·K) and the densities of its components—copper (632 kg/m³), silver (10,500 kg/m³), and kerosene oil (783 kg/m³).

**Fig. 8:**
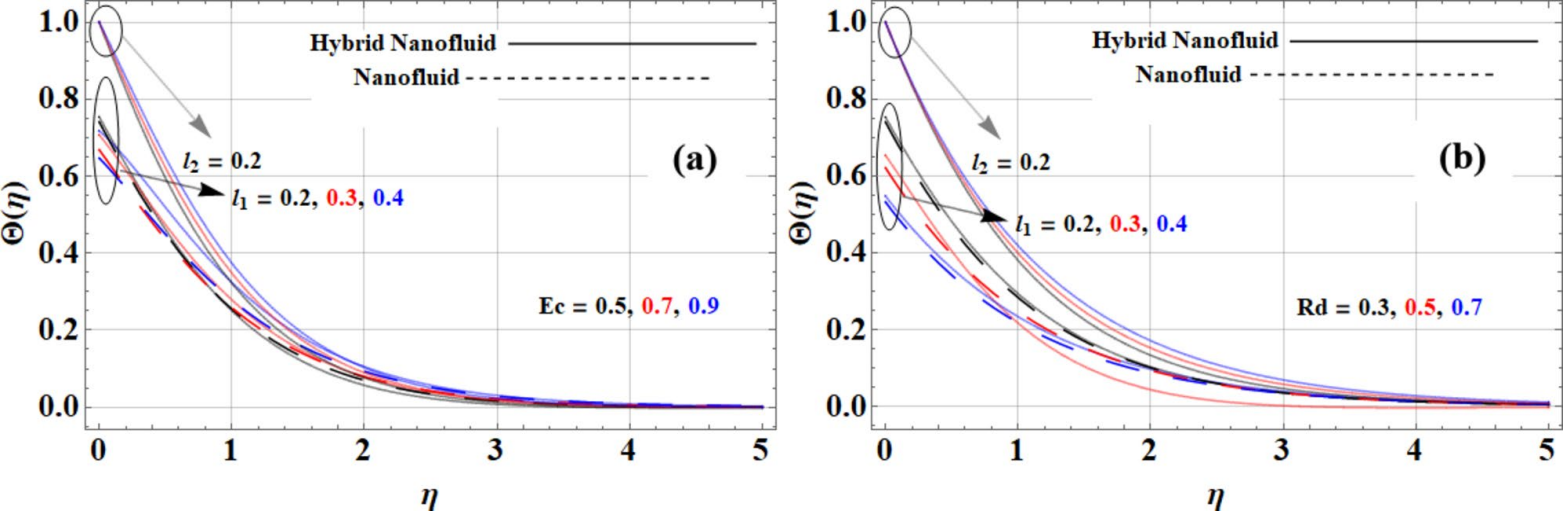
**(a)** Eckert number and **(b)** radiation parameter impact on temperature profiles.

The Eckert number and thermal radiation parameter significantly influence the velocity profiles of nanofluids under both slip and no-slip conditions are drawn in Fig. 8(a-b). As the Eckert number increases, the impact of viscous dissipation becomes more pronounced, particularly in no-slip conditions, leading to reduced velocities as kinetic energy contributes to heating rather than enhancing motion; for instance, in the *Cu + Kerosene Oil* nanofluid, the high thermal conductivity of copper (*765 W/m·K*) results in flatter velocity profiles. In slip conditions, while the effect of the Eckert number is mitigated, velocities may still decrease slightly due to viscous effects. Conversely, an increase in the thermal radiation parameter enhances heat transfer, promoting more uniform temperature distributions that can reduce viscosity and increase velocity profiles; this is especially evident in the Casson hybrid nanofluid (*Cu + GO + Kerosene Oil*), where graphene oxide’s exceptional conductivity (*5000 W/m·K*) significantly boosts fluid velocities. Overall, the interplay between the Eckert number and thermal radiation parameter determines the flow dynamics, with the Casson hybrid nanofluid demonstrating superior velocity performance due to enhanced heat transfer characteristics.

**Fig. 9:**
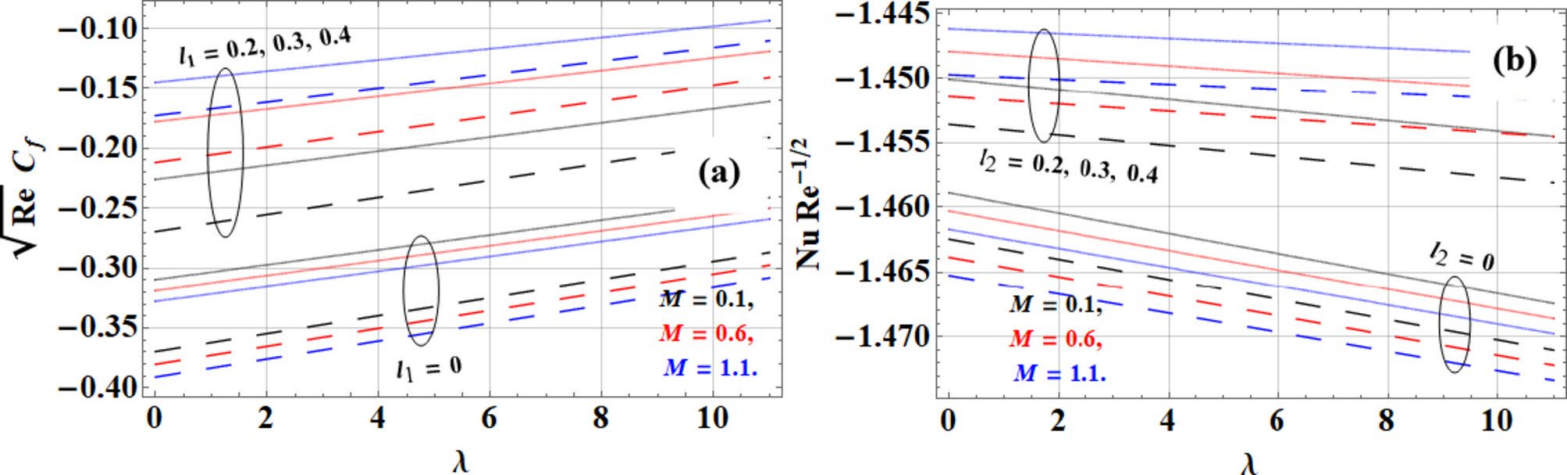
Magnetic parameter impact on **(a)** Skin friction and **(b)** Nusselt number.

The magnetic parameter increases skin friction and decreases the Nusselt number, but the effects differ in intensity due to slip and no-slip conditions as shown in Fig. 9(a-b). In slip conditions, the magnetic parameter increases skin friction by creating resistance (Lorentz forces), which is the cause of increasing friction. Additionally, the Nusselt number is lowered by the magnetic parameter, limiting convective heat transport. Because there is no slide at the boundary, there is less of an increase in skin friction, and improved convective heat transmission results in a less noticeable fall in the Nusselt number. The Casson hybrid nanofluid (*Cu + GO + Kerosene Oil*) performs best in maintaining heat transfer under these conditions, thanks to the high thermal conductivity of graphene oxide (*5000 W/m·K*), while *Cu + Kerosene Oil* exhibits better flow performance, reducing the impact on skin friction.

Heat enhancement is calculated percentage-wise for the Casson hybrid nanofluid, Casson hybrid nanofluid and simple nanofluid versus nanoparticles volume concentration in Table 4 as can be seen. Using the following formula:

$$\frac{{Kerosene{\text{ }}Oil{\text{ without nanoparticles}}}}{{Kerosene{\text{ }}Oil{\text{ }}with{\text{ nanoparticles}}}} \times 100={\text{Obtained Results,}}$$

$${\text{Obtained Results}} - {\text{100}}={\text{Enhancement Percentage}}{\text{.}}$$

Also, with the help of these calculations, it is determined that the Casson hybrid nanofluid has the best ability to transfer heat compared to other fluids for the recently considered model.

**Table 4:** Percent-wise heat enhancement of the Casson hybrid, Casson hybrid and simple nanofluid.

Nanoparticles volume concentration	Nusselt number	
${\phi _1},{\phi _2},{\phi _3}$	Casson hybrid	Casson hybrid
0.00	1.430082	1.430082
0.01	1.530530	1.500064
**% enhancement**	**6.562955%**	**4.6652676%**
0.02	1.590082	1.5775825
**% enhancement**	**10.062374%**	**9.3497804%**
0.03	1.7873963	1.67854898
**% enhancement**	**19.990770%**	**14.802486%**

## Conclusion

The analysis of Casson hybrid nanofluid (HNF) flow over a radially stretching sheet has significant applications in various fields, including industrial cooling, thermal management, and advanced engineering systems. Understanding the dynamic behaviour of HNF under time-dependent conditions is essential for optimizing heat transfer processes and enhancing the performance of cutting-edge technologies. In this study, we analytically investigated the time-dependent flow of Casson HNF, incorporating Copper and Graphene Oxide nanoparticles dispersed in Kerosene Oil. The impacts of magnetic fields, thermal radiation, suction and injection effects, and velocity and thermal slip conditions were comprehensively examined. The governing equations were transformed into nonlinear dimensionless differential equations and solved using the Homotopy Analysis Method (HAM). The core of study innovation also includes the extension of Darcy-Forchheimer theory along with the suction and injection effect and the demonstration of a 19.99% improvement in heat transfer performance, positioning Casson HNF as a promising solution for next-generation thermal management systems. The conclusion points provide a comprehensive summary of the recent research findings and their implications, demonstrating the significance of work in the field.

- The Casson hybrid nanofluid (*Cu + GO + Kerosene Oil*) exhibits superior temperature profiles due to the exceptional thermal conductivity of graphene oxide (*5000 W/m·K*), facilitating effective heat transfer and promoting uniform temperature distributions under various flow conditions.
- *The Cu + Kerosene Oil* nanofluid demonstrates the best velocity performance, primarily due to the high thermal conductivity of copper (*765 W/m·K*) and its favourable density (*632 kg/m³*), which allows for better flow characteristics, especially under slip conditions.
- Increasing the magnetic parameter dampens velocity profiles across all nanofluids due to the opposing Lorentz forces, leading to flattened velocity distributions, while simultaneously enhancing temperature profiles through improved thermal mixing, particularly in Casson hybrid and Casson hybrid nanofluids.
- The unsteady parameter adversely affects both velocity and temperature profiles, with higher values leading to decreased velocities and increased thermal gradients; however, the Casson hybrid nanofluid maintains better thermal performance due to its high thermal conductivity components.
- Suction significantly enhances velocity profiles by increasing fluid momentum, particularly in the *Cu + Kerosene Oil* nanofluid, while the Casson hybrid nanofluid excels in temperature uniformity due to effective mixing under suction conditions. Conversely, injection tends to reduce both velocity and temperature profiles.
- The Eckert number negatively impacts velocity profiles due to increased viscous dissipation, especially in no-slip conditions, whereas the thermal radiation parameter enhances heat transfer, resulting in improved velocities in both slip and no-slip scenarios, particularly for the Casson hybrid nanofluid.
- The results indicate that the Casson hybrid nanofluid (*Cu + GO + Kerosene Oil*) consistently demonstrates superior heat transfer performance, with up to a 6.55% enhancement in Nusselt number compared to the base nanofluid (*Cu + Kerosene Oil*) at a nanoparticle volume concentration of 0.01. Additionally, the Casson hybrid nanofluid (*Cu + Kerosene Oil*) shows a moderate improvement, with a maximum heat transfer enhancement of 4.06% at the same concentration.
- The Homotopy Analysis Method (HAM) employed in this study demonstrates high accuracy in predicting the velocity and temperature profiles of the nanofluids. Its flexibility in handling nonlinear differential equations allows for precise modelling of complex fluid behaviours under varying conditions, reinforcing the reliability of the results obtained.
- The findings underscore the potential of using nanofluids, particularly those incorporating graphene oxide, for applications requiring efficient heat transfer and flow dynamics, such as in cooling systems, thermal energy storage, and various industrial processes.
- Further investigations into the effects of varying concentrations of nanoparticles, different base fluids, and additional flow conditions are recommended to optimize the performance of nanofluids in real-world applications.

## Data Availability

The datasets used and/or analysed during the current study available from the corresponding author on reasonable request.

## Abbreviations

${B_0}$: Magnetic field (NA^-1^m^-1^)
u: Velocity Components (ms^-1^)
F (η): Dimensionless velocities
F_0_: Non-uniform inertia coefficient
$$T_\infty$$: Ambient temperature (K)
M: Magnetic parameter
r, z & φ: Cylindrical coordinates (m)
T: Fluid temperature (K)
T_w_: surface temperature
q_r_: Radiation heat flux (kgs^-3^)
k: Thermal conductivity (wm^-1^K^-1^)
${k_0}$: Permeability of porous space
$$\Theta (\mu)$$: Dimensionless temperature
Re: Reynold number
Pr: Prandtl number
Ec: Eckert number
$$C_f$$: Skin friction
Nu: Nusselt number

## Greek symbols

$\mu$: Dynamic viscosity (kgm^-1^s^-1^)
λ: Porosity Parameter
υ: Kinematic viscosity (m^2^s^-1^)
ρ: Density (kgs^-3^)
$\phi$: Volume fraction nanoparticles
$\rho c_p$: Specific heat capacity (jkg^-1^K^-1^)

## Subscripts

θ: Angle
f: Base Fluid
nf: Nanofluid
hnf: Ternary hybrid nanofluid
∞: Ambient condition
